# Targeting AI-2 quorum sensing: harnessing natural products against *Streptococcus suis* biofilm infection

**DOI:** 10.1186/s13567-025-01450-x

**Published:** 2025-02-04

**Authors:** Shuji Gao, Shuo Yuan, Yingying Quan, Wenjie Jin, Yamin Shen, Rishun Li, Baobao Liu, Yuxin Wang, Li Yi, Shaohui Wang, Xiaogai Hou, Yang Wang

**Affiliations:** 1 College of Animal Science and Technology, Henan University of Science and Technology, Luoyang, 471000 China; 2 Shanghai Veterinary Research Institute, Chinese Academy of Agricultural Sciences, Shanghai, China; 3 Henan Provincial Engineering Research Center for Detection and Prevention and Control of Emerging Infectious Diseases in Livestock and Poultry, Luoyang, 471003 China; 4 College of Life Science, Luoyang Normal University, Luoyang, 471934 China; 5 College of Agriculture/College of Tree Peony, Henan University of Science and Technology, Luoyang, Henan China

**Keywords:** *Streptococcus suis*, biofilm, natural products, quorum sensing, virulence

## Abstract

The biofilm acts as a protective layer for *Streptococcus suis* (*S. suis*), contributing to the development of drug resistance and chronic infections. Autoinducer 2 (AI-2) quorum sensing represents the primary regulatory pathway governing biofilm formation in *S. suis*. Consequently, targeting AI-2 quorum sensing to inhibit biofilm formation represents a promising strategy for preventing and managing drug resistance and chronic infections caused by *S. suis*. This study established a small natural product library by integrating commercial drug molecules with Chinese herbal medicine molecules. Consequently, two natural products, salvianolic acid A (SAA) and rhapontin (RH), which target *S. suis* AI-2 via quorum sensing, were identified. SAA and RH inhibit AI-2 synthesis through noncompetitive and competitive binding to S-ribosylhomocysteinase (LuxS). By inhibiting *S. suis* AI-2 quorum sensing, these compounds modulate the expression of adhesion genes and the synthesis of extracellular polysaccharides (EPS), reducing the adhesion ability of *S. suis* and ultimately inhibiting biofilm formation. Using LC‒MS/MS, we further analysed the impact of SAA and RH on the metabolic activity of *S. suis*, revealing the potential medicinal value of these compounds. Finally, the efficacy of SAA and RH against *S. suis* infection was validated in *Galleria mellonella* larvae, confirming their significant anti-infection effects.

**Supplementary Information:**

The online version contains supplementary material available at 10.1186/s13567-025-01450-x.

## Introduction

*Streptococcus suis* (*S. suis*) is a bacterium commonly located in the upper respiratory tract of pigs. Human infections with *S. suis* have emerged in Denmark, China, and Southeast Asia and are classified as zoonotic pathogens [1, 2]. *Streptococcus suis* serotype 2 is particularly notable for its high pathogenicity [3]. Since Grenier et al. first analysed the biofilm status of *S. suis* [4, 5], research on *S. suis* biofilms has increased significantly. A biofilm is a complex behavior of bacterial communities, typically forming a membrane-like structure composed of bacterial cells and extracellular polymeric substances such as extracellular polysaccharides (EPS) and extracellular DNA (eDNA) [6]. Any implanted nonnatural material can be a focal point for bacterial colonization and biofilm formation, potentially triggering an inflammatory response. Although the host immune response can reduce bacterial numbers, the aggregation properties of bacterial biofilms make it difficult for them to be wholly eradicated. Studies have shown that polymorphonuclear leukocytes (PMNs) can phagocytose only particles smaller than their size (approximately 10 μm) [7]. Therefore, biofilms of bacterial aggregates, approximately 12–15 μm in size, cannot be effectively phagocytosed by a single PMN. Even when multiple PMNs attack simultaneously, they can only successfully target aggregates less than 2–3 times their size [8, 9]. The surviving bacteria continue to grow, leading to recurrent clinical symptoms. While antibiotics are commonly used to treat *S. suis* infections, most bacteria within biofilms exist in a low metabolic state [10] or nongrowing state, fostering the formation of persisters. Consequently, biofilms enable *S. suis* to survive brief to lethal antibiotic concentrations, resulting in resistance to antimicrobial drugs [11]. Studies have demonstrated that the minimum inhibitory concentration (MIC) and minimum bactericidal concentration (MBC) of penicillin G and ampicillin in S. suis biofilms are 100–1000 times greater than those in planktonic *S. suis* [4]. Additionally, the horizontal transfer efficiency of resistance genes in biofilm bacteria is 700 times greater than that in planktonic bacteria [12].

Numerous factors influence the formation of bacterial biofilms. Biofilm formation is related to quorum sensing (QS), which is called “bacterial language”. QS is crucial for bacterial communication, allowing bacteria to produce and detect QS signal molecules, assess the composition and density of microorganisms in their environment, and dynamically adjust gene expression for optimal adaptation [13]. Pathogens utilize QS to regulate various life activities, including biofilm formation, cell metabolism, and motility [14–16]. Interfering with or blocking the QS pathway, thereby disrupting its regulatory functions, offers a novel approach for addressing bacterial drug resistance [17, 18]. Quorum-sensing inhibitors (QSIs) are substances that, without affecting normal bacterial activities, can block bacterial communication, interfere with, or inhibit QS. Unlike traditional antibiotics, QSIs target bacterial QS, effectively interfering with virulence factor production without promoting drug resistance [19]. This study identified the critical protein LuxS of *S. suis* autoinducer 2 (AI-2) QS as a target. Using virtual docking technology combined with in vitro experimental validation, we identified drugs with potential AI-2 QS inhibitory activity, providing a new direction for treating *S. suis* infections.

## Materials and methods

### Bacterial strains and culture conditions

*Streptococcus suis* HA9801 wild type and *Escherichia coli* (*E. coli*) BL21 (DE3) transformed with pET28-luxS and pET28-pfs have previously been described [20, 21]; *S. suis* were grown in Trypticase Soy Broth (TSB) at 37 ℃. The pET28-luxS (DE3) and pET28-pfs (DE3) strains were cultured at 37 °C in LB media supplemented with 50 μg/mL kanamycin (Shanghai Acmec Biochemical Technology Co., Ltd.). Natural product solutions (5 mg/mL or 20 mg/mL) (Beijing Solarbio Science & Technology Co., Ltd.) were stored at − 20 ℃.

### Virtual screening processes for quorum-sensing inhibitors (QSIs)

Initially, we integrated data from 5253 commercially available natural products from MedChemExpress and subsequently downloaded their 3D structures from PubChem (Additional file 1). Additionally, we employed TCMSP to identify the active ingredients of 5682 Chinese herbal medicines, including heat-clearing, detoxifying, and Hirakawa cough medicines (Additional file 2) [22]. High oral bioavailability (OB) ensures effective drug absorption, whereas drug likeness (DL) indicates the potential for the compound to be developed into new pharmaceuticals. Therefore, we screened drug components with OB ≥ 30% and DL ≥ 0.18 for further experimentation (Supplementary material 2) [23]. After removing duplicates and molecules with a molecular mass exceeding 500, 4126 small molecules were retained (Additional file 3). AutoDock Vina facilitated the energy minimization and conformational optimization of all small molecules, preparing them for subsequent screening. The LuxS protein was modelled on the basis of a framework developed in our previous study [20]. Initially, we conducted preliminary docking via AutoDock Vina, selected the top 10% of the docking results for further examination via AutoDock, and finally used SYBYL for the final screening round. We present 3D visualization of the molecular docking results and 2D interaction diagrams of protein‒ligand interactions using PyMOL and Discovery Studio Visualizer (version 2019, Dassault Systèmes, San Diego, USA), respectively.

### Molecular dynamics (MD) simulation and free energy landscapes

To process small-molecular files, we used acpype, subsequently generating itp and gro files [24]. In accordance with the established methodology, the Gromacs 2021.2 software package was utilized to simulate the binding stability of the protein‒ligand complex over a 100 ns timeframe [25]. Amber99-ildn.ff/tip3p was employed to generate protein topology files. The simulation box was subsequently defined, and the water model, ions, and balancing charges were in the specified sequence. After the NVT and NPT preequilibration phases were completed, subsequent analyses involved calculating the RMSD and RMSF. The free energy landscape (FEL) maps were projected onto the RMSD and radius of gyration (Rg) [26, 27], and visualization of the data was performed using Origin 2021.

### Minimal inhibitory concentration (MIC)

MIC determination was performed according to a previous method [28, 29]. A total of 100 μL of TSB was added to the 96-well plate; subsequently, 100 μL of mother liquor (Rhapontin (5 mg/mL), Salvianolic acid A (5 mg/mL), Tea polyphenol (20 mg/mL), and Phloridzin (20 mg/mL)) was added to the second column, and the liquid volume of each well was diluted in a double ratio from the second column. The liquid volume per well was ultimately 100 μL. Finally, 100 μL of diluted bacterial suspension (10^6^ CFU/mL) was added to each well and cultured for 24 h. For subsequent experiments in this study, both the MIC and sub-MIC concentrations of the natural products were utilized.

### Growth curves

The growth inhibition assay was conducted using a previously described method [30]. In summary, the culture medium was supplemented with natural products to achieve *S. suis* concentrations of 10^6^ CFU/mL, with final concentrations ranging from 1/16 MIC to MIC. These samples were incubated for 14 h. Every 2 h, samples were collected for optical density measurement at 600 nm.

### AI-2 quorum sensing inhibition assay

#### AI-2 production assay

In accordance with the methods of Han et al. [31], BL21 competent cells transformed with the plasmids pET28a-*luxS* and pET28a-*pfs* were used to express and purify the LuxS and Pfs proteins. SAH (1 mg/mL; Sigma, USA), the substrate for AI-2 synthesis, was added to 10 mM sodium phosphate buffer containing LuxS (1 mg/mL) and Pfs (1 mg/mL) and incubated at 37 ℃ for 15 min. Concurrently, natural products at subinhibitory concentrations were added. Proteins were subsequently removed via ultrafiltration using a 10 000 Da cut-off filter (Millipore, USA). The resulting reaction products were then diluted 20-fold in 100 mM phosphate-buffered saline (PBS) (pH 7.2, 0.1 mM EDTA). The diluent was combined with a 5 mM solution of 5,5-dithiobis-2-nitrobenzoic acid in identical sodium phosphate buffer at a 2:1 ratio. After a 15-min reaction at 37 °C, the absorbance was recorded at 412 nm.

#### Enzyme activity inhibition assay

SRH (the LuxS substrate) was prepared according to a previous protocol [32]. A 1 mg/mL solution of SAH and 1 M HCl was reacted in boiling water for 20 min. The SRH solution was diluted to 4 mM with 100 mM sodium phosphate buffer. The effects of natural products on LuxS activity were indirectly measured using the Ellman method [25]. Different concentrations of SRH (0–1200 μM) were added to sodium phosphate buffer containing LuxS (1 mg/mL). The experimental group included natural products at gradient concentrations (final volume 100 μL), which were incubated for 15 min at 37 ℃. Then, the mixture was combined with 100 μL of Ellman’s reagent and incubated for an additional 30 min at 37 ℃. The OD_412_ was recorded, and the homocysteine concentration was determined by interpolating this value into the standard curve [32]. The production of homocysteine per unit time represented the enzyme activity of LuxS.

### Biofilm inhibition assays

#### Determination of biofilm

*Streptococcus suis* (10^6^ CFU/mL) was premixed with the natural product mixture and added to a 96-well cell culture plate. After a 48-h incubation, the suspended bacteria were washed with PBS, fixed with 95% methanol, and stained with 0.1% (w/v) crystal violet for 15 min. Subsequently, the samples were washed with PBS to remove excess crystal violet. After drying, the crystal violet solution was dissolved in 95% ethanol, and the absorbance at 595 nm was measured using an automatic microplate reader [25, 33].

#### Visualization of biofilms by scanning electron microscopy

Briefly, *S. suis* cultures (1 × 10^6^ CFU/mL in TSB supplemented with natural products) were seeded in 12-well plates with a cell climbing slide at the bottom. These plates were incubated for 24 h without agitation. The slides on which the cells climbed were rinsed with PBS. After the supernatant was removed, the slides were fixed in 2.5% glutaraldehyde. The slides were dehydrated through graded ethanol solutions [34]. Following air drying, the biofilms were examined using SEM (JSM-5610LV; JEOL, Japan).

### Extracellular polysaccharide inhibition assay

*Streptococcus suis* (1 × 10^6^ CFU/mL) was premixed with various concentrations of SAA (20, 40, or 80 μg/mL) or RH (10, 20, or 40 μg/mL) and cultured for 48 h. The culture mixture was centrifuged to obtain the supernatant. Three volumes of ethanol were mixed with the supernatant and incubated at 4 °C for 12 h. Afterward, the mixture was centrifuged under the same conditions such that the residue was resuspended in 1 mL of ddH_2_O. The resuspended solution was mixed with phenol (6%) and concentrated sulfuric acid at a ratio of 2:1:5 and incubated in a water bath at 60 °C for 20 min [32]. The OD_490_ was measured. The inhibition rate of EPS was calculated as follows: inhibition rate (%) = 100 × (drug-addition group−control group)/control group.

### qRT‒PCR

Total RNA extraction was performed with the TRIzol method, after which cDNA was synthesized via reverse transcription [35]. Specific primers targeting 16S rRNA served as internal controls. The comparative critical threshold (2^−ΔΔCΤ^) method was applied to analyse the amplification data. The primer sequences are provided in Table 1.

**Table 1:** **Primers used for the quantitative RT‒PCR analysis**

Genes	Primer sequence
*16SrRNA*-1	GTTGC GAACG GGTGA GTAA
*16SrRNA-*2	TCTCA GGTCG GCTAT GTATC G
*fbps*-1	AACCA TCTTG CCAGG CTCCA C
*fbps*-2	CAGTT CAGAA GCCGT ATCCC GAC
*gdh*-1	CACCT TTACC ACCGC CGATT G
*gdh*-2	GGAAA TGTTC AAGTC AACCG TGG
*gapdh*-1	CTTGG TAATC CCAGA ATTGA ACGG
*gapdh*-2	TCATA GCAGC GTTTA CTTCT TCAGC
*ef*-1	TCCAA TCACA GATCC AGATA GCG
*ef*-2	CTGAC CCATT TGGAC CATCT AAG
*mrp*-1	CAGGT AACAT CAGAA TCACC ACTTT T
*mrp*-2	AAGTT TTGTT TGAGC ATCCT CTATA GC

### Cell assays

#### CCK-8 assay

Human laryngeal epidermoid carcinoma (HEp-2) cells (1 × 10^4^ cells/well) were seeded in 96-well plates. The cells were allowed to grow for 18 h in DMEM containing 10% fetal bovine serum (FBS) (Jianglai Biology, Shanghai). Following this initial incubation period, the medium was carefully removed, and the cells were washed with PBS to remove any dead cells. Next, the medium was replaced with 100 μL of DMEM containing 2% FBS and various concentrations of SAA (20, 40, or 80 μg/mL) or RH (10, 20, or 40 μg/mL). The plates were then incubated for 24 h. Then, 10 μL of CCK-8 was added to each well, and the plates were further incubated for 1 h. Finally, the absorbance at 450 nm (OD_450_) was measured using a microplate reader [36]. The results were analysed to assess cell viability, and the treated groups were compared with the control group to determine the toxicity of SAA and RH to HEp-2 cells.

#### Adherence assay

A previous protocol was applied with slight modifications [37]. HEp-2 cells were cultured in DMEM (containing 10% FBS). A total of 1 × 10^5^ cells per well were inoculated in 24-well plates and cultured at 37 ℃ with 5% CO_2_ for 16 h, after which the monolayer Hep-2 cells were washed with PBS. One milliliter of DMEM (containing 2% FBS) was added to each well, followed by the addition of 1 × 10^6^ *S. suis*. The drug treatment group received either 20, 40, or 80 μg/mL SAA or 10, 20, or 40 μg/mL RH. After the plates were incubated for 2 h, the culture medium was discarded, and floating *S. suis* was removed with PBS. After treatment with 100 μL of trypsin for 1 min, the cells were resuspended in PBS, plated on TSB agar, and cultured at 37 ℃ for 24 h. The assays were performed in triplicate.

### *Galleria mellonella* larvae protection assay

In accordance with Nadya et al.’s method for establishing a *Galleria mellonella* (*G. mellonella*) larval model [38], there were 10 larvae in each group, and 20 μL of *S. suis* (1 × 10^8^ CFU/mL) was used to infect the right posterior proleg of each larva. In addition, SAA (20, 40, or 80 μg/mL) or RH (10, 20, 40 μg/mL) treatment was performed 2 h after bacterial infection. Within 72 h of injection, the survival rate of the larvae was recorded every 12 h.

### Metabolomic analysis

*Streptococcus suis* was treated with SAA or RH for 8 h. Equivalent cells were quenched with liquid nitrogen and collected by centrifugation at 8500 × *g* at 4 ℃ for 15 min. The metabolites were extracted with 1 mL of cold solvent (chloroform/methanol, 1/5, v/v). The cells were lysed by sonication at 30 W for 5 min, followed by centrifugation at 12 000 × *g* for 15 min at 4 °C. A total of 500 μL of the supernatant was filtered through a 0.22 μm filter and transferred to a 2 mL microtube. Finally, the samples were analysed via an LC‒MS/MS system. Six biological replicates were performed per sample.

LC‒MS analysis was performed using a five-stage technique described previously [39]. Mobile phases A and B were ultrapure water and methanol (Sigma). The optimized gradient profile was as follows: 0–8 min (5% B), 8–18 min (35% B), 18–22 min (35% B), 22–28 min (90% B), 28–30 min (50% B), and 30–32 min (0% B). Electrosspray ionization (ESI) mode was employed with a source spray voltage of + 3800 V ( +) or − 3100 V (−). The capillary temperature was 320 ℃. The range of mass full scan mode was 65–995 m/z. The sheath gas flow rate was 45 Arb. The auxiliary gas flow rate was 15 Arb.

Metabolomic data were analysed using MetaboAnalyst 6.0 [40]. A Sankey bubble diagram was created using an online platform for data analysis [41].

### Statistical analysis

Assays were performed in triplicate, and the means ± standard deviations were calculated. The data were analysed via one-way and two-way analysis of variance (ANOVA) with SPSS 22.0.

## Results

### Virtual screening results

**Figure 1:**
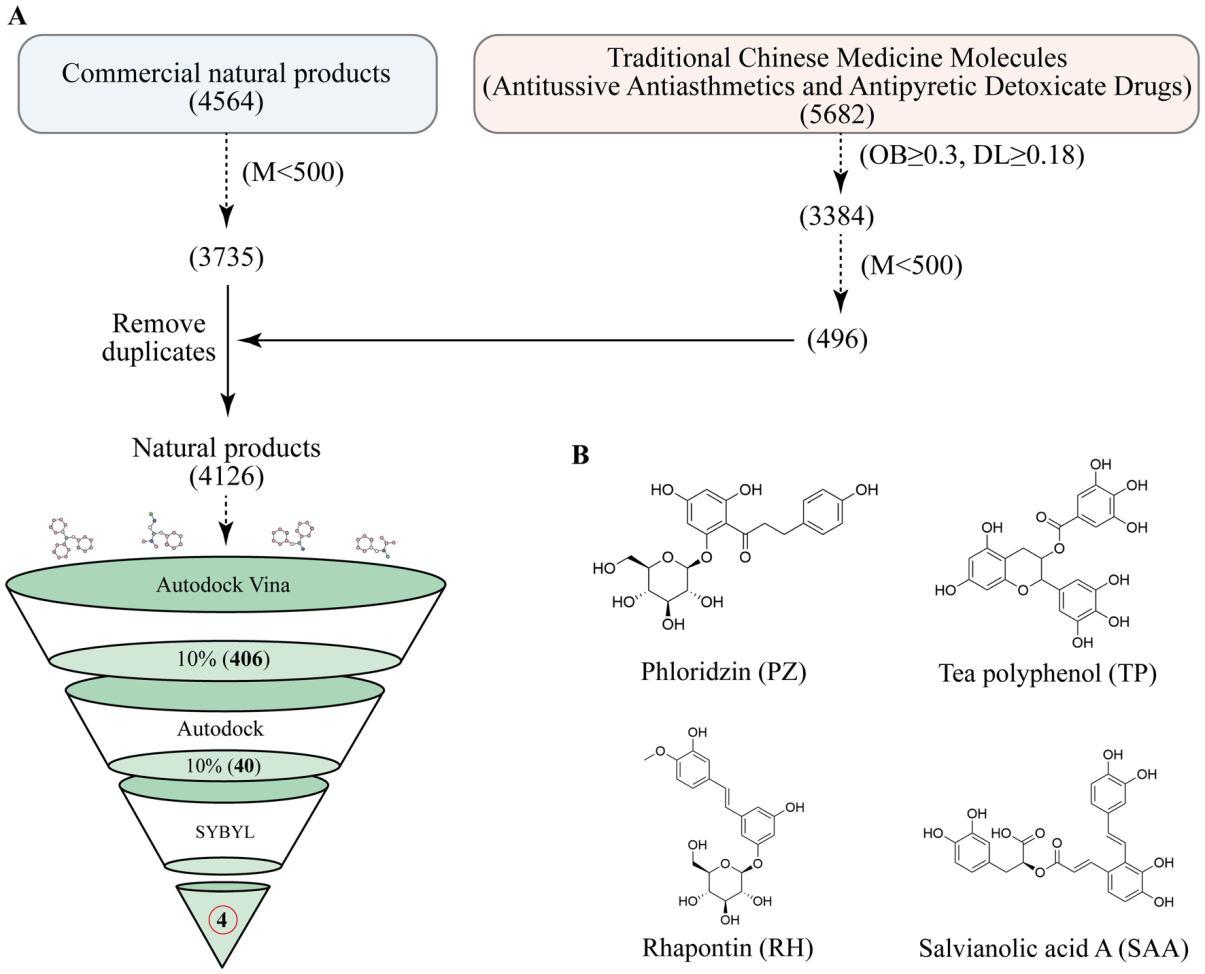
**Virtual screening of the QSIs from the natural product library**. **A** Screening process. **B** Screened compounds.

The compounds underwent docking with the LuxS protein, with details on the compounds detailed in Supplementary material 4. Following Autodock Vina fast screening, a total of 4066 compounds were filtered. The top 10% were subsequently subjected to further scrutiny through AutoDock, and on this basis, SYBYL was used to verify the top 10% (Figure 1A). Ultimately, the compounds with the highest four docking scores, specifically Rhapontin (RH), Salvianolic acid A (SAA), Tea polyphenol (TP), and Phloridzin (PZ), were chosen for subsequent experiments (Figure 1B).

### Molecular docking and MD simulation

**Figure 2:**
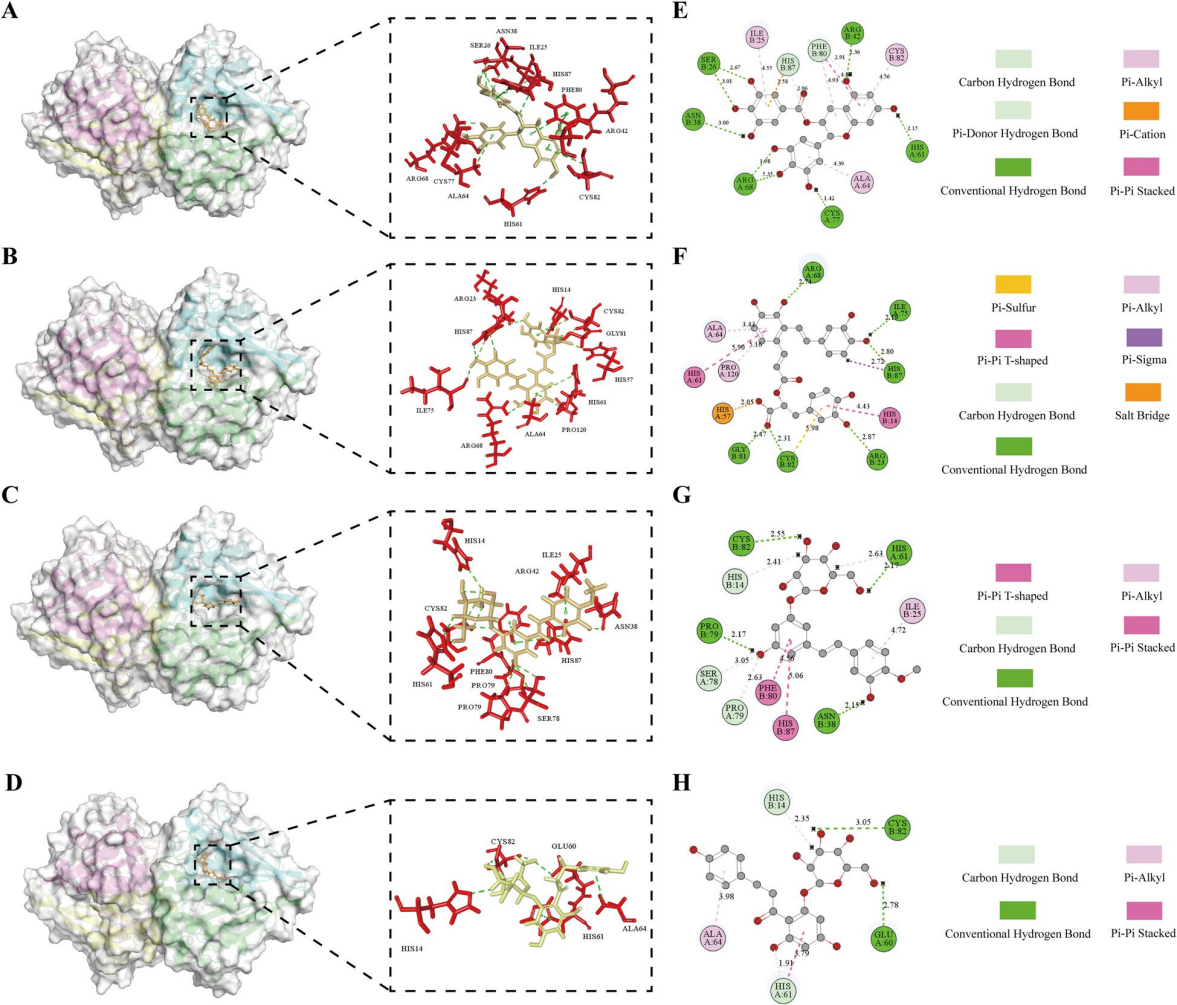
**Visualization of the virtual screening results**. The molecular docking results are shown in 3D images (**A**–**D**) and 2D images (**E**–**H**). **A**, **E** LuxS and TP docking results. **B**, **F** LuxS and SAA docking results. **C**, **G** LuxS and RH docking results. **D**, **H** LuxS and PZ docking results.

PyMOL was used to visualize the molecular docking results (Figure 2A–D), whereas Discovery Studio visualized the protein‒ligand interactions in two dimensions (Figures 2E–H). These four natural products demonstrate various intermolecular interactions with LuxS, including hydrogen bonds (conventional hydrogen bonds, carbon‒hydrogen bonds, pi donor hydrogen bonds, and salt bridges), electrostatic interactions (pi-cation and salt bridges), hydrophobic forces (e.g., pi‒pi stacking, pi‒pi T-shaped, pi‒sigma, and pi‒alkyl), and other interactions (notably pi‒sulfur). As illustrated in Figure 2, TP primarily forms with LuxS through conventional hydrogen bonds and pi‒alkyl interactions (Figure 2E). SAA exhibits the most diverse interaction profile with LuxS, encompassing pi-sulfur, pi-pi T-shaped, pi-alkyl, pi-sigma, salt bridge, carbon‒hydrogen bonding, and conventional hydrogen bonding. Notably, the pi‒sulfur interaction, a noncovalent bond, plays a crucial role in the stability of the cysteine/aromatic configuration (Figure 2F) [42]. The interaction of RH with LuxS involves conventional and carbon‒hydrogen bonds. Additionally, hydrophobic interactions, including pi‒pi stacking, pi‒alkyl bonds, and pi‒pi T‒shaped bonds, are formed between the benzene rings of RH and LuxS (Figure 2G). PZ interacts with LuxS through conventional hydrogen bonds, carbon‒hydrogen bonds, pi‒alkyl interactions, and pi‒pi stacking. These bonds are facilitated by the benzene ring of PZ and the amino acid residues of LuxS (Figure 2H). The interactions of these four compounds with LuxS are characterized predominantly by carbon‒hydrogen and conventional hydrogen bonds, which are fundamental to molecular binding stability [43, 44]. Additionally, other noncovalent bonds, while weaker than covalent bonds, contribute significantly to binding stability through their collective strength, which is vital for the interaction between small molecules and proteins [45, 46]. The distance of certain conventional hydrogen bonds between the small molecules and LuxS is less than 3 Å, suggesting a substantial likelihood of forming robust hydrogen bonds [43, 47]. Moreover, CH/π hydrogen bonds, including pi‒pi stacking and pi‒alkyl interactions, are observed between the natural products and LuxS. These bonds are pivotal in protein‒ligand recognition and signal transduction systems [48].

**Figure 3:**
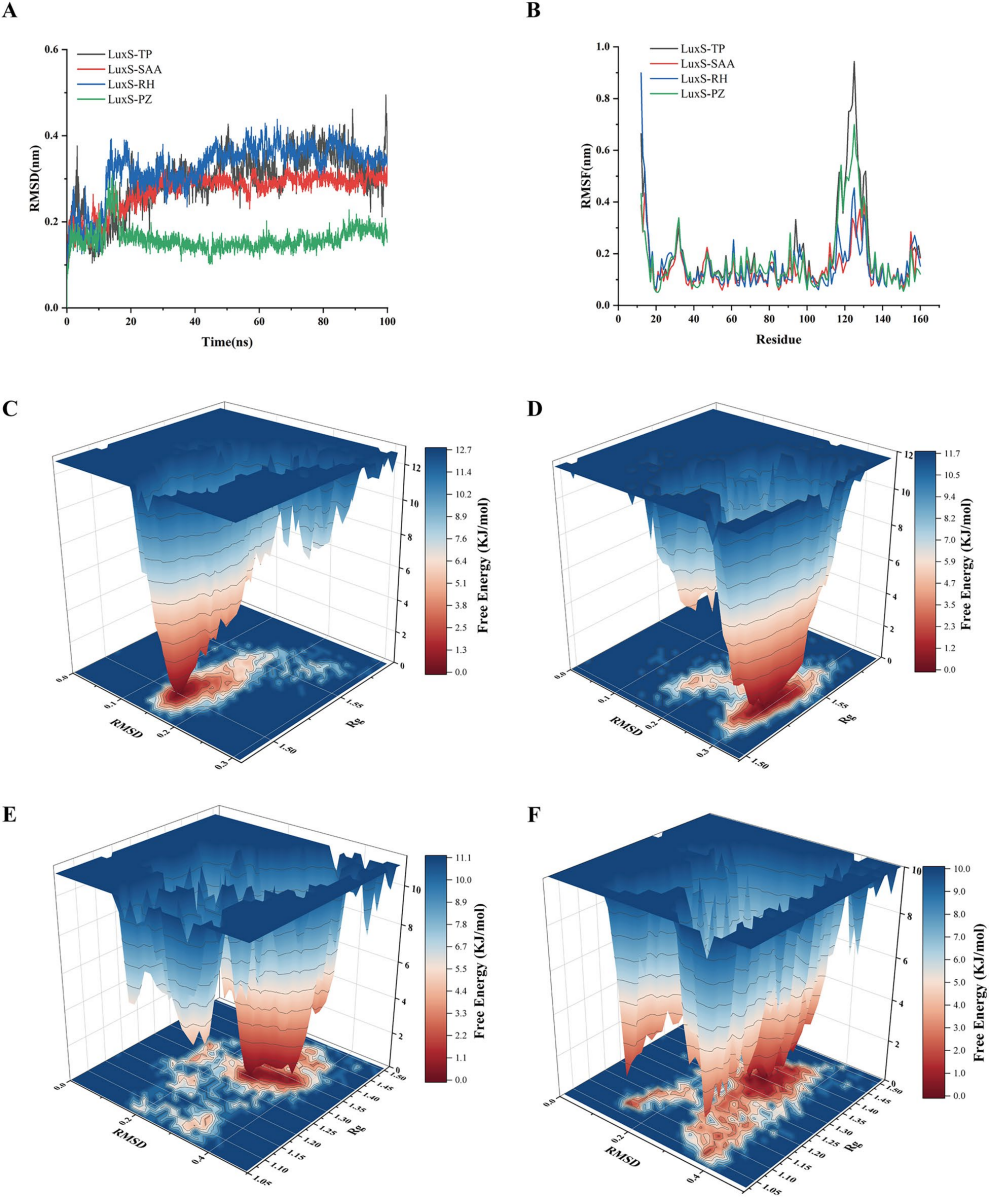
**Molecular dynamics simulation analysis (A, B) and free energy landscape analysis (C–F)**. **A** RMSD distribution map. **B** RMSF distribution map. The black line represents the complex of LuxS and TP, the red line represents the complex of LuxS and SAA, the blue line represents the complex of LuxS and RH, and the green line represents the complex of LuxS and PZ. **C** LuxS-TP complex. **D** LuxS-SAA complex. **E** LuxS‒RH complex. **F** LuxS-PZ complex.

The binding stability of the enzyme‒substrate complex, assessed at 100 ns intervals, was quantified via the root mean square deviation (RMSD) and root mean square fluctuation (RMSF). The RMSD, a crucial parameter in molecular dynamics (MD) simulations, gauges the protein's balance and flexibility during docking and monitors the distance between the protein backbone and its atoms. Minor fluctuations and lower RMSD values indicate a stable enzyme‒substrate connection [49]. Throughout the 100 ns simulation, the RMSD values of LuxS, in conjunction with the four natural products, fluctuated within a range of ± 0.5. The most stable complexes were LuxS-PZ and LuxS-SAA, which exhibited final RMSD values of approximately 0.15 nm and 0.28 nm, respectively (Figure 3A). The fluctuation range of their RMSD was within ± 0.1 Å. In comparison, LuxS-TP demonstrated significant fluctuations in binding, with a final RMSD value of approximately 0.4 nm, indicating weaker stability. RMSF analysis revealed changes in the protein's flexible regions after binding to various substrates and identified fluctuations in protein residues during the MD simulation. Lower RMSF values are associated with greater stability [24]. The results indicated that LuxS-SAA and LuxS-RH were relatively stable overall. Larger residue fluctuations were observed in the 110–130 amino acid region of LuxS-TP and LuxS-PZ, suggesting weaker stability (Figure 3B). Despite not having the highest docking score among the four compounds, LuxS-SAA exhibited excellent stability during binding. Free energy landscapes (FELs) depict protein stability in terms of Gibbs free energy [50, 51]. These were represented as 3D FEL maps, with RMSD and Rg illustrated as a contour map at the base of each FEL plot to reveal different conformational states (Figures 3C–F). The stability of the complex can be assessed through its low potential energy and central conformational space. LuxS-TP is the least stable complex and is characterized by one low-energy basin (Figure 3C). LuxS-SAA displays three central conformational spaces and two low-energy basins (Figure 3D). LuxS-RH has two central conformational spaces and two low-energy basins (Figure 3E). The most structurally stable complex, LuxS-PZ, features three central conformational spaces and two low-energy basins (Figure 3F).

### Effects of natural products on *S. suis* AI-2 quorum sensing

**Figure 4:**
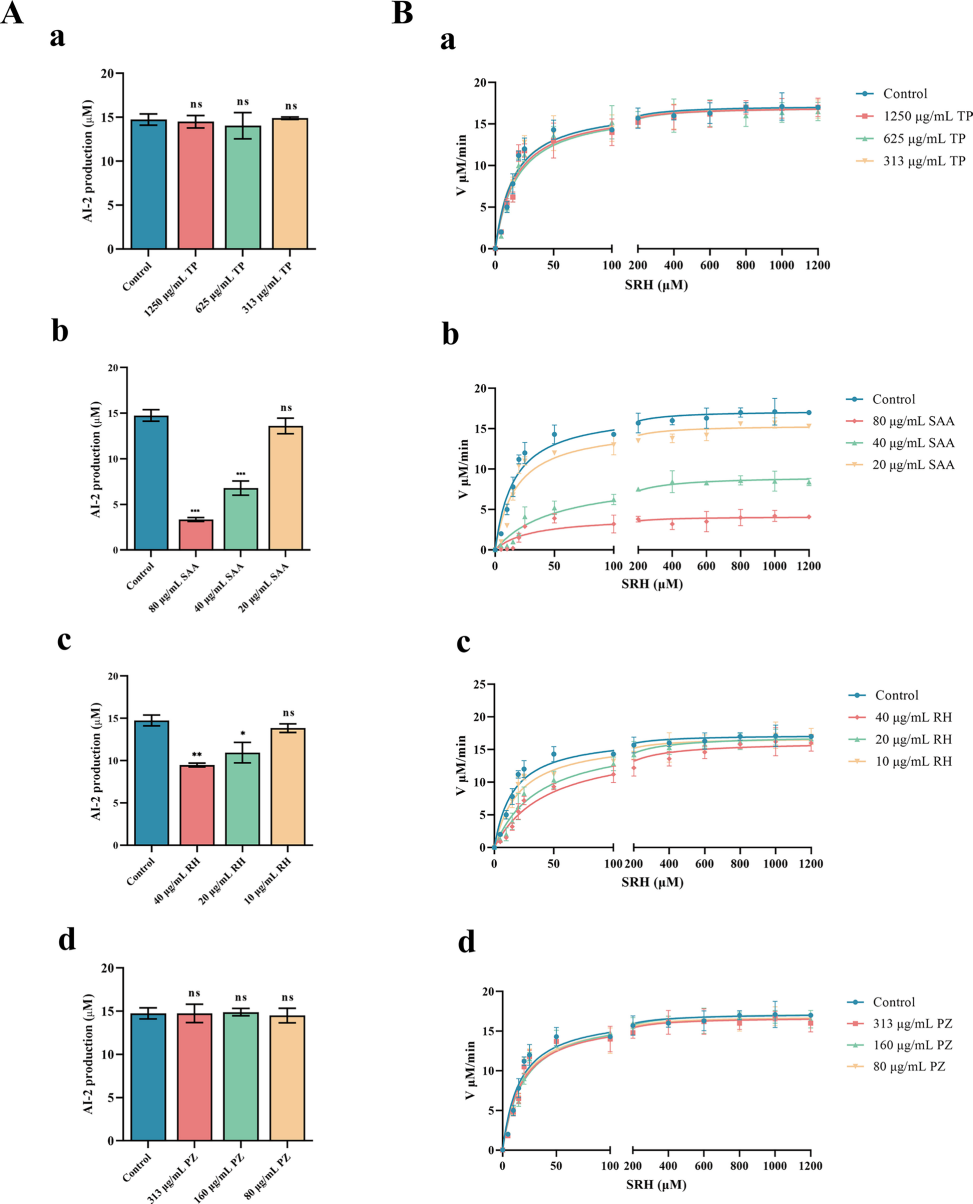
**Inhibitory effects of natural products on AI-2 QS**. **A** Inhibitory effects of TP (a), SAA (b), RH (c) and PZ (d) on AI-2 synthesis. **B** Inhibitory effects of TP (a), SAA (b), RH (c) and PZ (d) on LuxS activity. *P < 0.05, ^**^P < 0.01, and ^***^P < 0.001 indicate significant differences compared with the untreated control.

In conjunction with virtual simulations, we confirmed the in vitro inhibitory effects of the four natural products on the LuxS protein. Initially, we expressed the Pfs and LuxS proteins (Additional file 5), followed by their interaction with the substrate SAH to produce homocysteine and DPD (the precursor of AI-2). We quantified the AI-2 content by measuring homocysteine production. A comparison of the AI-2 content at the end of the reaction allowed us to assess the impact of natural products on AI-2 synthesis preliminarily. The drug concentrations used here were consistent with the subsequent experimental concentrations and did not affect *S. suis* growth (Table 2) (Additional file 6). Among the four screened natural products, only SAA and RH significantly inhibited AI-2 synthesis (Figure 4A, panels a–d). We conducted an enzyme activity inhibition test to further evaluate the inhibitory effects of SAA and RH on the LuxS protein. We observed the effects of adding gradient concentrations of SRH to natural products. TP and PZ did not have inhibitory effects on the LuxS protein (Figure 4B, panels a and d). Interestingly, AI-2 synthesis in the SAA group did not increase with increasing SRH concentration, suggesting a noncompetitive inhibitory effect of SAA on LuxS (Figure 4B, panels b and c). Conversely, AI-2 synthesis in the RH group increased with increasing substrate concentration, whereas the inhibitory effect of RH decreased, indicating a competitive inhibitory impact of RH on LuxS.

**Table 2:** **MICs of TP, SAA, RH and PZ against *S. suis***

	TP	SAA	RH	PZ
MIC (μg/mL)	2500	320	160	1250

### Effects of natural products on *S. suis* biofilms

**Figure 5:**
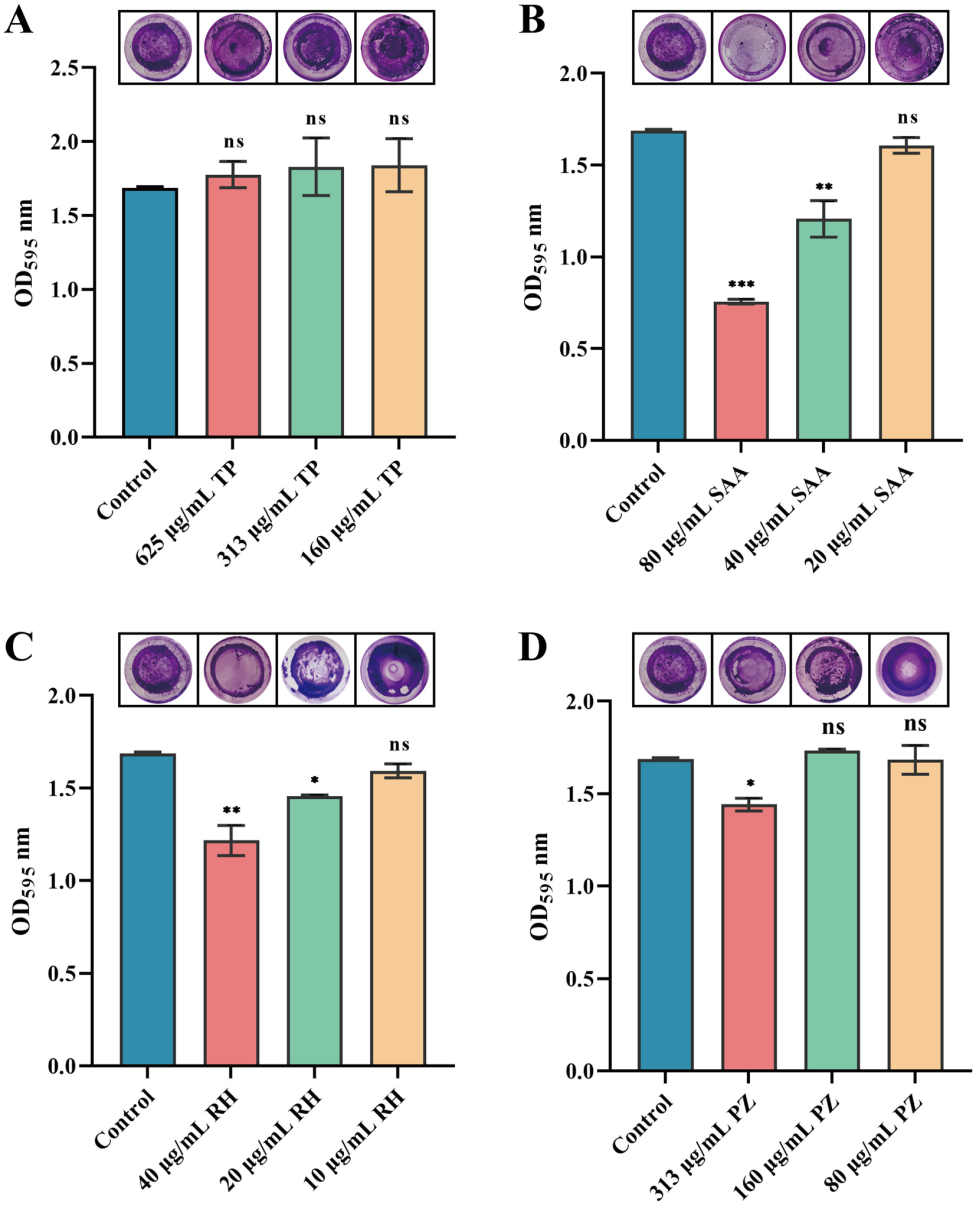
***Streptococcus suis* biofilms under the action of natural products (A–D)**. 24-H Biofilm content of *S. suis* treated with TP (**A**), SAA (**B**), RH (**C**) or PZ (**D**). *P < 0.05, ^**^P < 0.01, and ^***^P < 0.001 indicate significant differences compared with the untreated control.

AI-2 QS plays a crucial role in *S. suis* biofilm formation. Through virtual screening and in vitro AI-2 synthesis inhibition tests, we identified two naturally active components, SAA and RH, which may be promising; however, their specific effects still need further verification. Therefore, we further determined the impact of four natural products on *S. suis* biofilm formation at concentrations that did not affect bacterial growth. The natural products were incubated with *S. sui* for 24 h, and the content of crystal violet was determined to determine the inhibitory effects of the natural products on *S. suis* biofilms. The results showed that TP and PZ had no significant inhibitory effect on *S. suis* biofilm formation (Figures 5A and D). In contrast, SAA and RH at 1/8 MIC to 1/4 MIC significantly inhibited *S. suis* biofilms (Figures 5B and C). These findings, combined with the AI-2 inhibition results, indicate that TP and PZ have no medicinal value in treating *S. suis* biofilms. Therefore, only SAA and RH at 1/16 MIC to 1/4 MIC were used for follow-up experiments. We then observed the microscopic inhibition of *S. suis* biofilms by SAA and RH. A SAA and RH at 1/8 MIC to 1/4 MIC caused *S. suis* to disperse and prevented aggregation (Figure 5A). Additionally, the morphology of *S. suis* was affected, with the cellular structure collapsing and becoming sunken (Figure 5A, red dotted arrow). Furthermore, the filamentous substances between *S. suis* decreased (Figure 5A, green dotted arrow), suggesting that SAA and RH may impact *S. suis* biofilm matrix production, thereby inhibiting biofilm formation.

**Figure 6:**
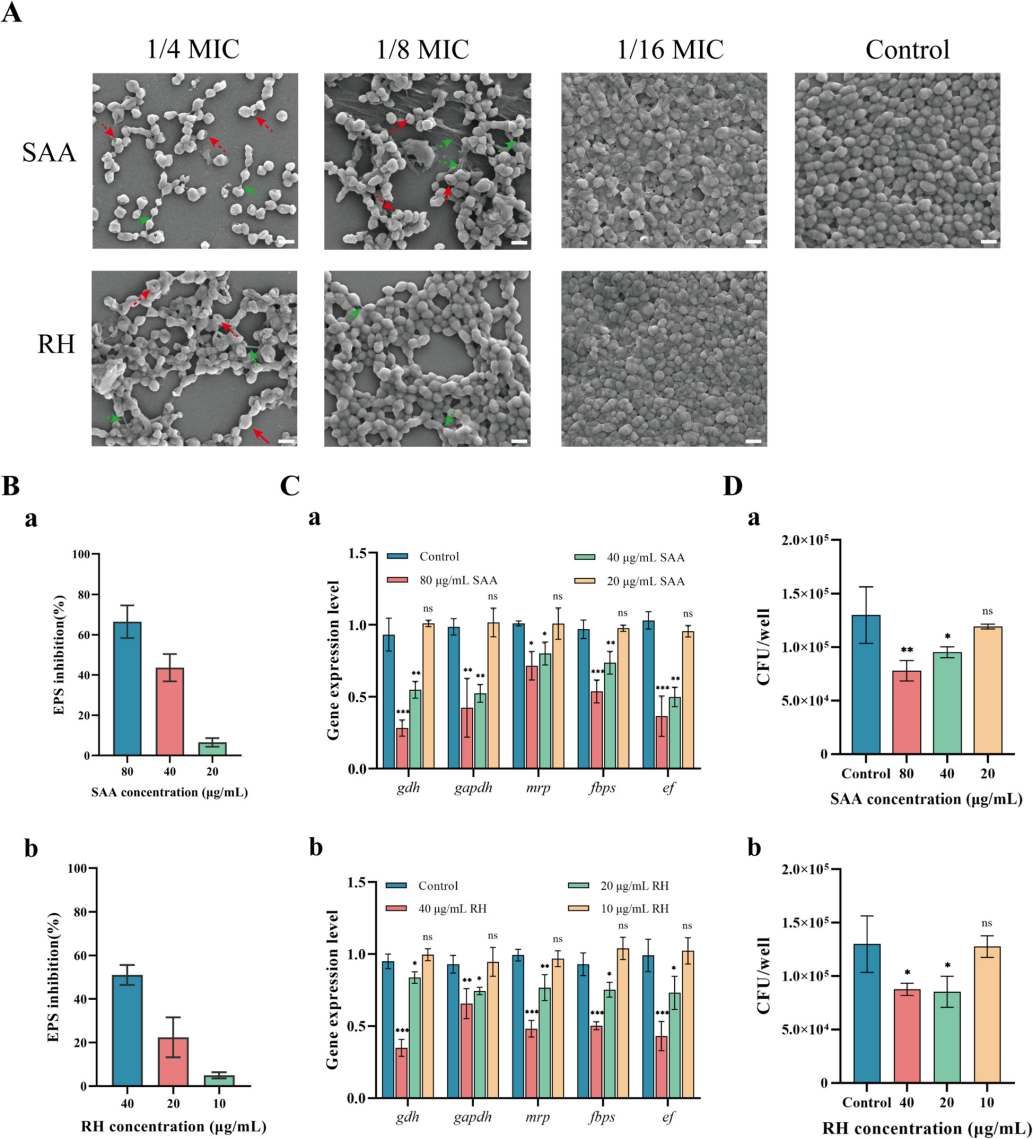
**Natural products weaken the adhesion ability of *S. suis***. **A** Scanning electron microscopy results. **B** Inhibition effects of SAA or RH on *S. suis* EPS. **C** Inhibition of adhesion gene expression in *S. suis* by SAA or RH. **D** SAA or RH inhibits the adhesion of *S. suis* to HEp-2 cells. *P < 0.05, ^**^P < 0.01, and ^***^P < 0.001 indicate significant differences compared with the untreated control.

The bacterial biofilm matrix mainly comprises polysaccharides and proteins, with EPS accounting for a significant proportion [52]. EPSs are negatively charged and bind to cationic antibiotics (such as tobramycin) [53, 54], reducing the diffusion rate of antibiotic molecules in biofilms and ultimately affecting their ability to kill bacteria. This is one of the reasons why biofilm bacteria are challenging to eradicate. Additionally, biofilm formation is influenced by the adhesion ability of bacteria. Therefore, we measured the EPS content and the expression levels of adhesion genes in *S. suis*. The results showed that SAA and RH at 1/8 MIC to 1/4 MIC could significantly inhibit the synthesis of EPS in *S. suis* (Figure 6B). In addition, SAA and RH significantly inhibited the expression of adhesion-related genes (Figure 6C). We further assessed bacterial adhesion ability to determine the effects of natural products on bacterial adhesion. We first measured the cytotoxicity of SAA and RH. The results indicated that SAA at concentrations of 1/4 MIC to MIC was toxic to cells, whereas RH was cytotoxic at concentrations of 1/2 MIC to MIC (Additional file 7). Compared with those in the control group, the number of bacteria adhering to HEp-2 cells significantly decreased in response to SAA and RH (Figure 6D). These results showed that SAA and RH could inhibit the synthesis of EPS and weaken the adhesion ability of *S. suis*, which is consistent with observations from a scanning electron microscope.

### Effects of natural products on *S. suis* infection

**Figure 7:**
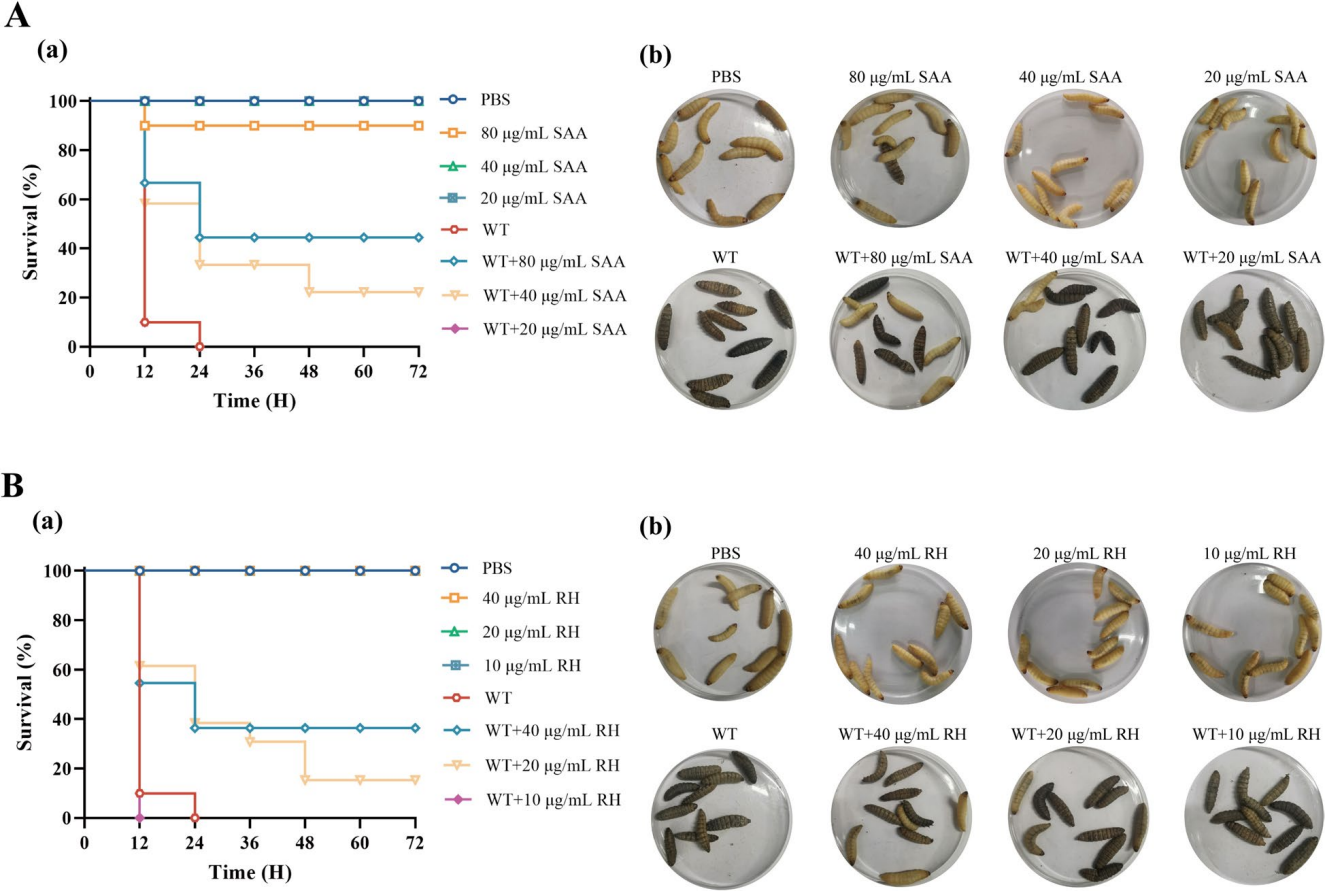
**Protective effects of SAA and RH on *Galleria mellonella* larvae infected with* S. suis***. **A** Survival rate of *Galleria mellonella* treated with SAA. **B** Survival rate of *Galleria mellonella* treated with RH.

We used the *G. mellonella* larval model to conduct drug protection experiments and confirm the protective effects of SAA and RH. There were 10 larvae in each group, with the PBS injection group serving as the negative control. The results revealed that the *G. mellonella* larvae injected with *S. suis* alone died within 24 h (Figure 7A). In contrast, both SAA and RH had protective effects. The protective effect of 40 μg/mL SAA was weak. Seventy-two hours after infection, the larval survival rate remained at 20%, whereas 80 μg/mL SAA increased the larval survival rate to 50% (Figure 7A, panels a and b). The effect of RH was slightly weaker than that of SAA. The results revealed that the survival rate of larvae remained at 20% after treatment with 20 μg/mL RH and increased to 40% after treatment with 40 μg/mL RH (Figure 7B, panels a and b). Similarly, neither SAA nor RH at 1/16 MIC had a therapeutic effect. These results suggest that SAA and RH have the potential to treat *S. suis* infection.

### Effect of natural products on *S. suis* metabolic activity

**Figure 8:**
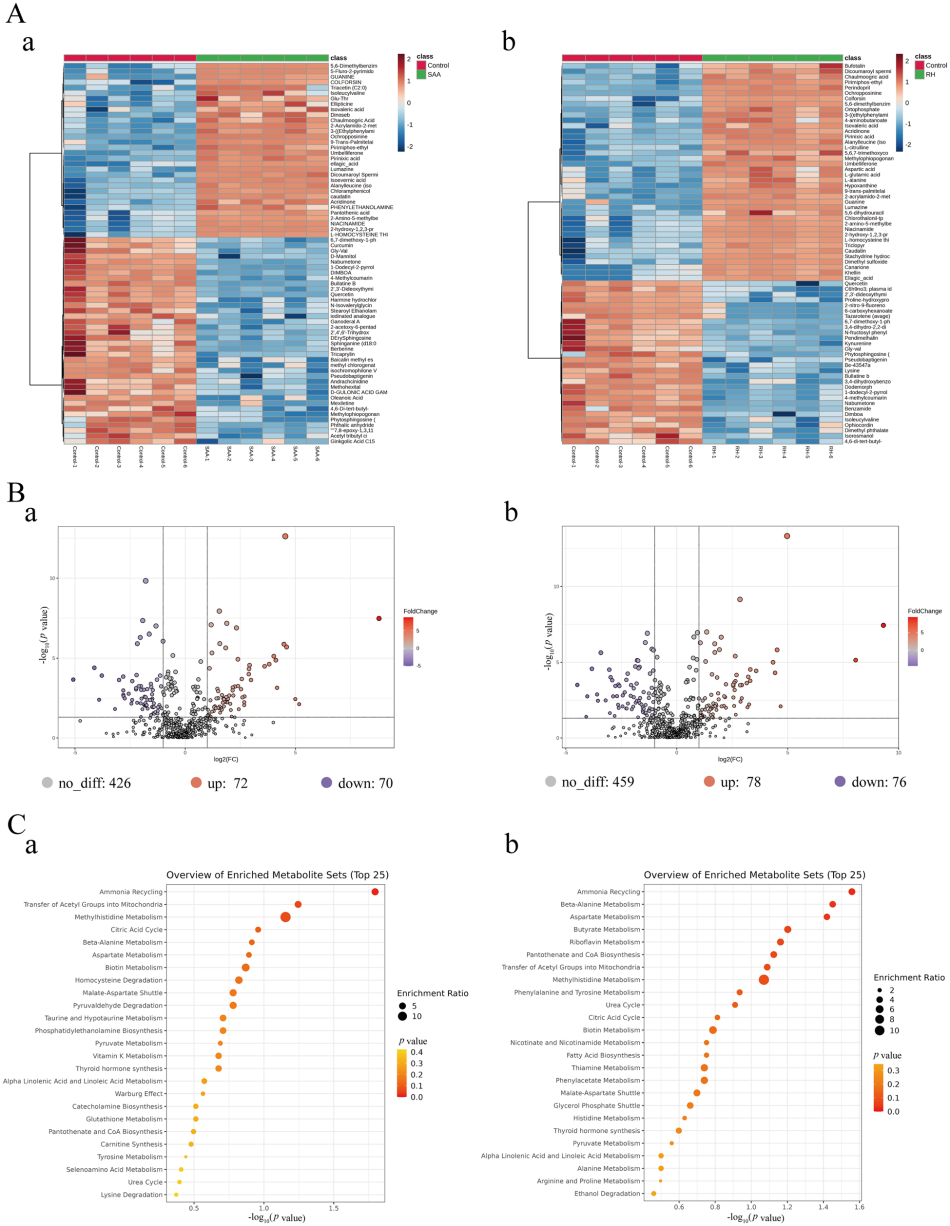
**Effects of SAA and RH on the metabolic activities of *S. suis***. **A** LC‒MS metabolite heatmap under the action of SAA (a) and RH (b). **B** Volcano map of differential metabolism under the action of SAA (a) and RH (b). **C** Enrichment analysis of differential pathways affected by SAA (a) and RH (b).

**Figure 9:**
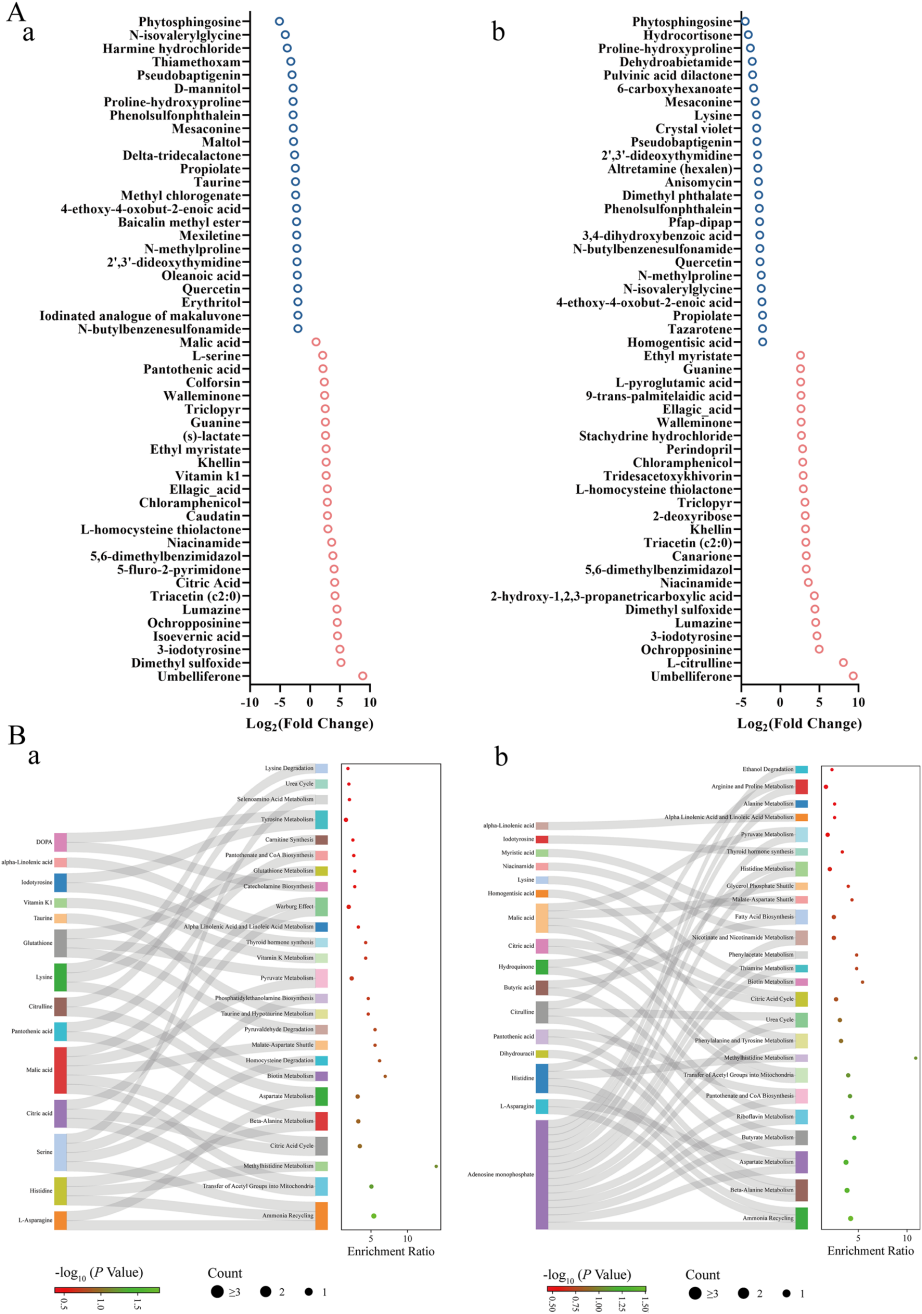
**Effects of SAA and RH on the metabolic pathways of *S. suis***. **A** Representative metabolite alterations in *S. suis* upon SAA (a) and RH (b) treatment. **B** Analysis of metabolic pathway enrichment induced by SAA (a) and RH (b).

To further explore the medicinal value of SAA and RH, we used nontargeted metabonomics to analyse their effects on the metabolic activity of *S. suis* (Figures 8 and 9). SAA upregulated 72 metabolites and downregulated 70 metabolites of *S. suis* (Figure 8B, panel a), whereas RH upregulated 78 metabolites and downregulated 76 metabolites (Figure 8B, panel b). Differentially abundant metabolite enrichment analysis revealed that ammonia recovery and the metabolism of several amino acids (such as methylhistidine metabolism, β-alanine metabolism, and aspartic acid metabolism) in *S. suis* were affected by SAA and RH. In the SAA-treated group, aerobic metabolic activity, including the citric acid cycle (tricarboxylic acid cycle, TCA cycle), was significantly regulated (Figure 8C, panel a). Similarly, the citric acid cycle was also affected in the RH treatment group, indicating that both drugs may influence the aerobic metabolism of *S. suis* (Figure 8C, panel b). To further analyse differential metabolic activity, we listed the top 50 differentially abundant metabolites (Figure 9A, panels a and b). In the SAA treatment group, the content of L-homocysteine thiolactone was increased (Figure 9A, panel a), which is consistent with our conclusion that SAA interferes with *S. suis* AI-2 QS and contributes to methionine and purine metabolism [55], leading to blockage of the homocysteine metabolism pathway and ultimately leading to the accumulation of L-homocysteine thiolactone. Additionally, the levels of malic acid and citric acid, which are related to the citric acid cycle, were significantly increased (Figure 9A, panel a), indicating that SAA may accelerate the *S. suis* citric acid cycle in addition to targeting LuxS. We further visualized the relationships between differentially abundant metabolites and metabolic pathways. Many metabolic activities associated with malic acid and citric acid, such as pyruvate metabolism, malate-aspartate shuttle, and the citric acid cycle, were affected (Figure 9B, panel a). These findings support our hypothesis that SAA promotes the aerobic metabolic activity of *S. suis* but is disadvantageous to biofilm formation [56]. Similarly, the results of the RH processing group were consistent with those of the SAA. L-homocysteine thiolactone also accumulated in the RH group, and metabolic activities associated with malic acid and citric acid were observed after RH treatment (Figure 9B, panel b). Additionally, RH significantly affected adenosine monophosphate-related amino acid metabolism, such as aspartic acid metabolism, histidine metabolism, and alanine metabolism. These results suggest that SAA and RH target LuxS and have great potential for medicinal development.

## Discussion

The biofilms of microorganisms are closely related to their pathogenicity. Biofilms can protect microorganisms from attack by the host system and antibiotics, thereby increasing their survival time in the host and improving their adhesion and invasiveness [37, 57]. The antibacterial activity of antibiotics generally relies on the ability of the drug to reach the target within the bacterial cell at a sufficient concentration. A biofilm, which is composed of microorganisms and their secretions, forms a membrane-like structure through aggregation. This structure represents an independent pharmacokinetic microchamber, the third pharmacokinetic microcompartment, which is distinct from the bloodstream (first compartment) and interstitial fluid (second compartment). Therefore, the drug is depleted when it reaches the biofilm and is hindered by the biofilm structure and the slow growth of bacterial cells within the biofilm, and the accessibility of bacterial targets decreases [58, 59]. The actual concentration of antibiotics may not reach the required systemic treatment concentration [53, 60]. A better therapeutic effect can be achieved by interfering with biofilm formation and then treating with antibiotics. Rifampicin combined with other antibiotics can treat staphylococcal biofilm infection because it can act as an antibiofilm agent, penetrate the biofilm matrix, and target nongrowing cells [61]. Therefore, it is necessary to inhibit bacterial biofilm formation.

The relationship between bacterial QS and biofilm regulation has been widely studied [62, 63]. The formation of biofilms is a cooperative group behavior involving the survival of bacterial populations in their extracellular matrix. QS synchronizes gene expression with population density and coordinates the biofilm transition when reaching a threshold level to adapt to a new lifestyle [64]. Evidence from different bacteria shows that QS activation occurs within established biofilms, regulating their maturation and degradation [65, 66]. *Streptococcus suis* AI-2 QS can regulate the expression of adhesion genes and promote biofilm formation [21, 67]. Therefore, developing new drugs that target bacterial QS is a promising antibacterial biofilm strategy [68, 69]. However, the extensive size of the global drug library has rendered traditional drug search strategies inefficient in meeting clinical needs. Therefore, applying computational functions such as data calculation, storage, graphic processing, and prediction to the screening and design of QSIs can reduce the randomness of traditional compound search methods, reduce experimental costs, accelerate research and development, provide researchers with a visual representation of theoretical concepts, and facilitate intuitive understanding and explanation of experimental results.

**Figure 10:**
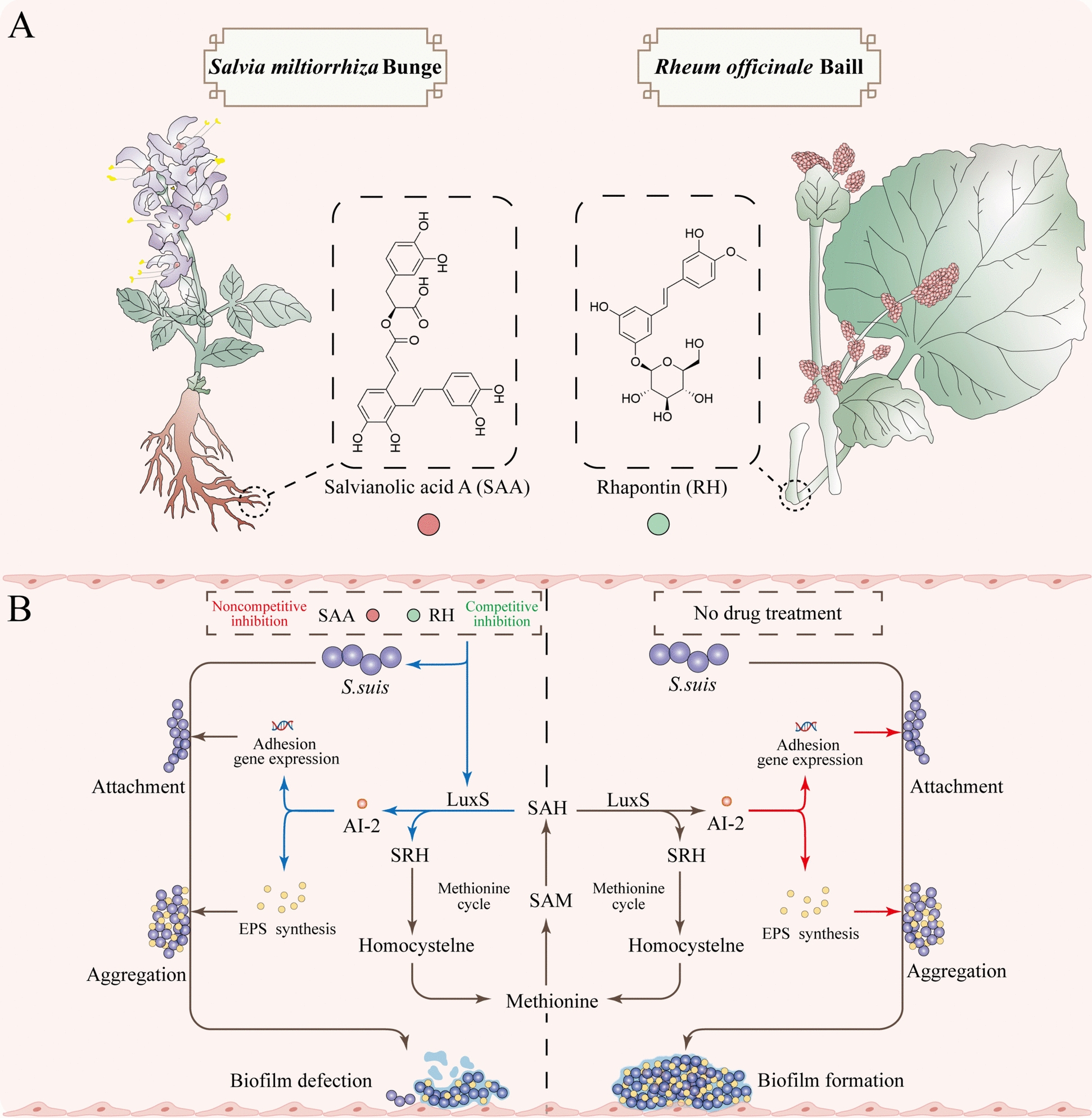
**Mechanism by which SAA and RH inhibit *S. suis* biofilm formation**. **A** SAA is present in the roots of *Salvia miltiorrhiza* Bunge. RH is present in the dried roots or rhizomes of *Rheum officinale* Baill. **B** SAA and RH inhibit the production of AI-2 via noncompetitive and competitive inhibition of LuxS, respectively, and further inhibit EPS synthesis and the expression of adhesion genes, resulting in *S. sui*s biofilm defects. SAM: S-adenosylmethionine; SAH: S-adenosylhomocysteine; SRH: S-ribosylhomocysteine; LuxS, S-ribosylhomocysteinase.

In this study, considering that *S. suis* is a respiratory tract colonization bacterium, we chose a Chinese herbal medicine with heat-clearing and detoxification properties and Hirakawa cough. We combined them with a commercial small-molecule library (Additional file 3). On this basis, we selected LuxS, the critical protein of *S. suis* AI-2 QS, as the target protein and obtained four natural products, namely, TP, SAA, RH, and PZ (Figures 1, 2, 3), through virtual screening. On this basis, we demonstrated the inhibitory effects of these four natural products on AI-2 signalling molecules through in vitro experiments and biofilm inhibition tests, ultimately screening SAA (from the root of *Salvia miltiorrhiza* Bunge) [70, 71] and RH (from the dried root or rhizome of *Rheum officinale* Baill) [72] as two natural small molecules with LuxS-targeting effects for follow-up research (Figures 4 and 5). Further experimental results revealed that SAA and RH can effectively inhibit EPS synthesis and adhesion in *S. suis* and have therapeutic effects. SAA and RH influence LuxS by targeting amino acid residues, including arginine (ARG), isoleucine (ILE), and alanine (ALA). Specifically, these compounds interact with LuxS through carbon‒hydrogen bonds and are pi‒pi T-shaped. Notably, both compounds engage with the 61st and 87th positions of the histidine (HIS) and the 82nd position of cysteine (CYS) in LuxS. SAA forms pi‒pi T-shaped bonds with HIS61, whereas RH establishes carbon‒hydrogen bonds with the same residue. Conversely, for HIS87, the interactions are reversed, which may explain why SAA inhibits LuxS noncompetitively, whereas RH results in competitive inhibition. Ultimately, the inhibition of LuxS by SAA and RH disrupts AI-2 synthesis, weakens the EPS synthesis and adhesion ability of *S. suis*, and ultimately inhibits biofilm formation (Figure 10). However, the potential of SAA and RH is much greater. Therefore, we analysed the bacterial metabolites treated with SAA and RH via LC‒MS/MS. The results showed that both SAA and RH affected the homocysteine metabolism of *S. suis*, inhibiting intracellular L-homocysteine thiolactone metabolism (Figure 8A, a, b). When LuxS synthesizes AI-2 signalling molecules, it is also a corner of the methionine metabolic cycle [73]. Therefore, it is unsurprising that LuxS activity is blocked and that l-homocysteine thiolactone of the methionine metabolic pathway is affected. In addition, beta-alanine metabolism, ammonia recycling, and aspartate metabolism were also significantly affected. Interestingly, SAA and RH significantly affected the aerobic metabolism of S. suis, which is dominated by the citric acid cycle (Figure 8B, panels a and b). The metabolic activity of bacteria is closely related to biofilm formation. Biofilm bacteria are often in a state of low metabolism, which is a self-protective state where antibiotics are less effective [74]. SAA and RH significantly activate the citric acid cycle of *S. suis* to prevent its metabolic activity from slowing, thus inhibiting its “dormancy”. These findings suggest that SAA and RH may be used with antibiotics to maintain the killing effect of antibiotics by hindering the slowdown of bacterial metabolism. More interestingly, glucose-induced aerobic metabolism is associated with AI-2 QS, which has been confirmed in *E. coli* [75]. This raises another question: are the effects of SAA and RH on the aerobic metabolism of *S. suis* due to their direct effects on bacterial aerobic metabolism or indirect effects through AI-2 QS? Analysing this issue is essential for expanding the medicinal potential of SAA and RH.

A drug molecule library was constructed, and two small Chinese medicine molecules targeting *S suis* AI-2 QS were screened. SAA and RH inhibit the activity of LuxS, thereby hindering the synthesis of AI-2. This further hinders AI-2 QS-associated activities, inhibits the adhesion ability of *S suis*, and ultimately inhibits biofilm formation. Excitingly, SAA and RH significantly affect the metabolic activity of *S suis*, indicating that SAA and RH have potential drug functions beyond targeting AI-2 QS. In addition, the drug molecular library established in this study may also be used for screening other drug targets in *S suis*.

## Supplementary Information

**Additional file 1.** **Commercial natural products**.

**Additional file 2.** **Traditional Chinese medicine molecules**.

**Additional file 3.** **Integrated natural product information**.

**Additional file 4.** **Virtual docking results.**

**Additional file 5.** **SDS‒PAGE analysis of total cellular proteins and purified proteins from *E. coli* cells**. Lane 1: Total protein from *E. coli* BL21 containing pET28a. Lane 2: SDS‒PAGE analysis of total cellular proteins containing the expression plasmids pET28a-*pfs*. Lane 3: Elution of the Pfs-purified fusion protein from the affinity column.

**Additional file 6.** **Growth curve of *S. suis* in the presence of natural products.** (A) Growth of *S. suis* under the action of TP (A), SAA (B), RH (C) and PZ (D).

**Additional file 7.** **Drug toxicity of natural products**.

## Data Availability

The datasets generated during and analyzed during the study are available from the corresponding author on reasonable request.

## Abbreviations

AI-2: autoinducer 2
QS: quorum sensing
SAA: salvianolic acid A
RH: rhapontin
TP: tea polyphenol
PZ: phloridzin
QS: quorum sensing
LuxS: S-ribosylhomocysteinase
EPS: extracellular polysaccharides
MIC: minimal inhibit concentration
PBS: phosphate buffered saline
SEM: scanning electron microscopy
FBS: fetal bovine serum
RMSD: root mean square deviation
RMSF: root mean square fluctuation
FEL: free energy landscapes
TCA cycle: tricarboxylic acid cycle
