# Supplementary material for: Targeting AI-2 quorum sensing: harnessing natural products against Streptococcus suis biofilm infection
Source: Vet Res. 2025 Feb 4;56:26. doi: 10.1186/s13567-025-01450-x (PMC11796197; doi:10.1186/s13567-025-01450-x)
Supplement: Supplementary file 5 — Additional file 5. SDS‒PAGE analysis of total cellular proteins and purified proteins from E. coli cells. Lane 1: Total protein from E. coli BL21 containing pET28a. Lane 2: SDS‒PAGE analysis of total cellular proteins containing the expression plasmids pET28a-pfs. Lane 3: Elution of the Pfs-purified fusion protein from the affinity column. [file 13567_2025_1450_MOESM5_ESM.docx]

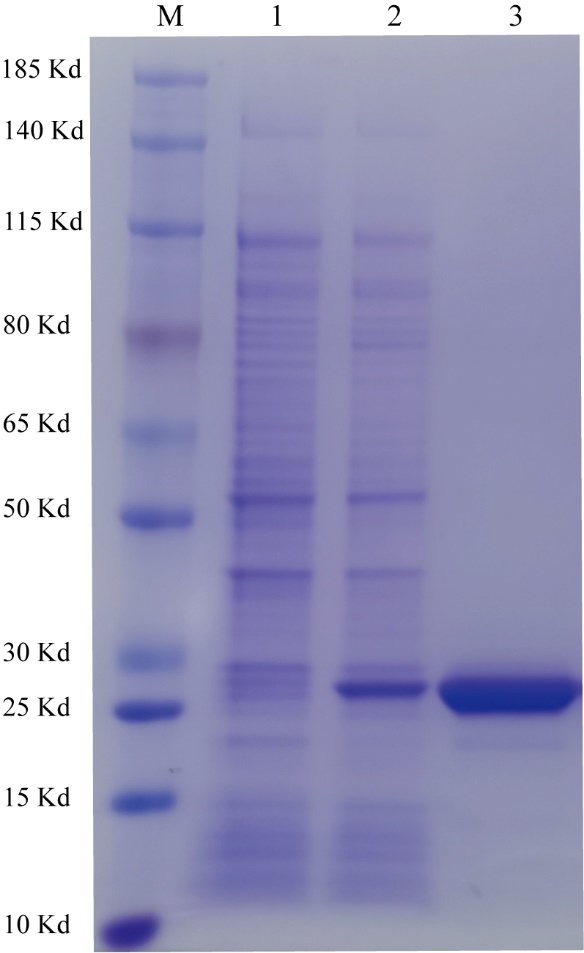


**Additional file 5. SDS-PAGE analysis of total cellular proteins and purified fusion proteins from *E. coli* cells.** Lane 1: Total cellular proteins from *E. coli* BL21 containing pET28a. Lane 2: SDS-PAGE analysis of total cellular proteins containing expression plasmids pET28a-*pfs*. Lane 3: Elution of the Pfs purified fusion protein from the affinity column.


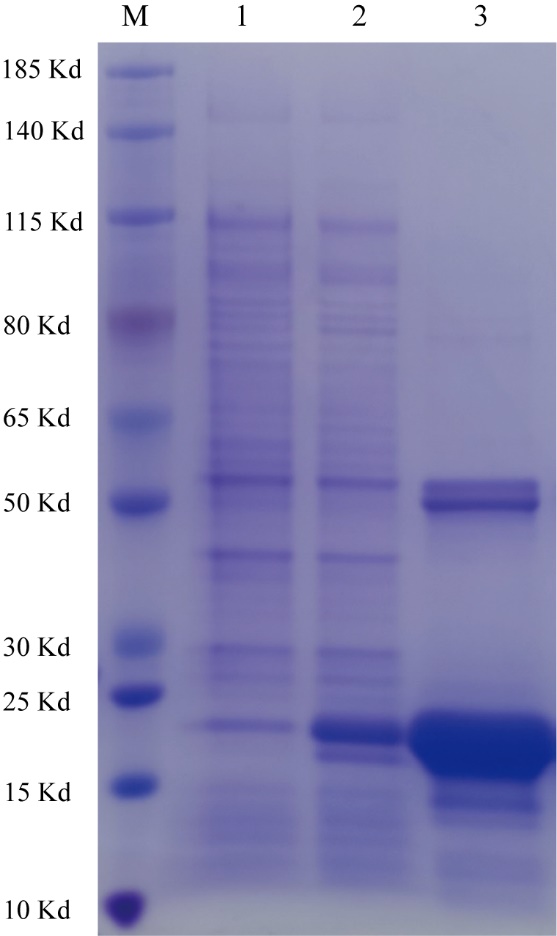


Figure S2. SDS-PAGE analysis of total cellular proteins and purified fusion proteins from *E. coli* cells. Lane 1: Total cellular proteins from *E.coli* BL21 containing pET28a. Lane 2: SDS-PAGE analysis of total cellular proteins containing expression plasmids pET28a-*luxS*. Lane 3: Elution of the LuxS purified fusion protein from the affinity column.
