# Supplementary material for: Targeting AI-2 quorum sensing: harnessing natural products against Streptococcus suis biofilm infection
Source: Vet Res. 2025 Feb 4;56:26. doi: 10.1186/s13567-025-01450-x (PMC11796197; doi:10.1186/s13567-025-01450-x)
Supplement: Supplementary file 6 — Additional file 6. Growth curve of S. suis in the presence of natural products. (A) Growth of S. suis under the action of TP (A), SAA (B), RH (C) and PZ (D). [file 13567_2025_1450_MOESM6_ESM.docx]

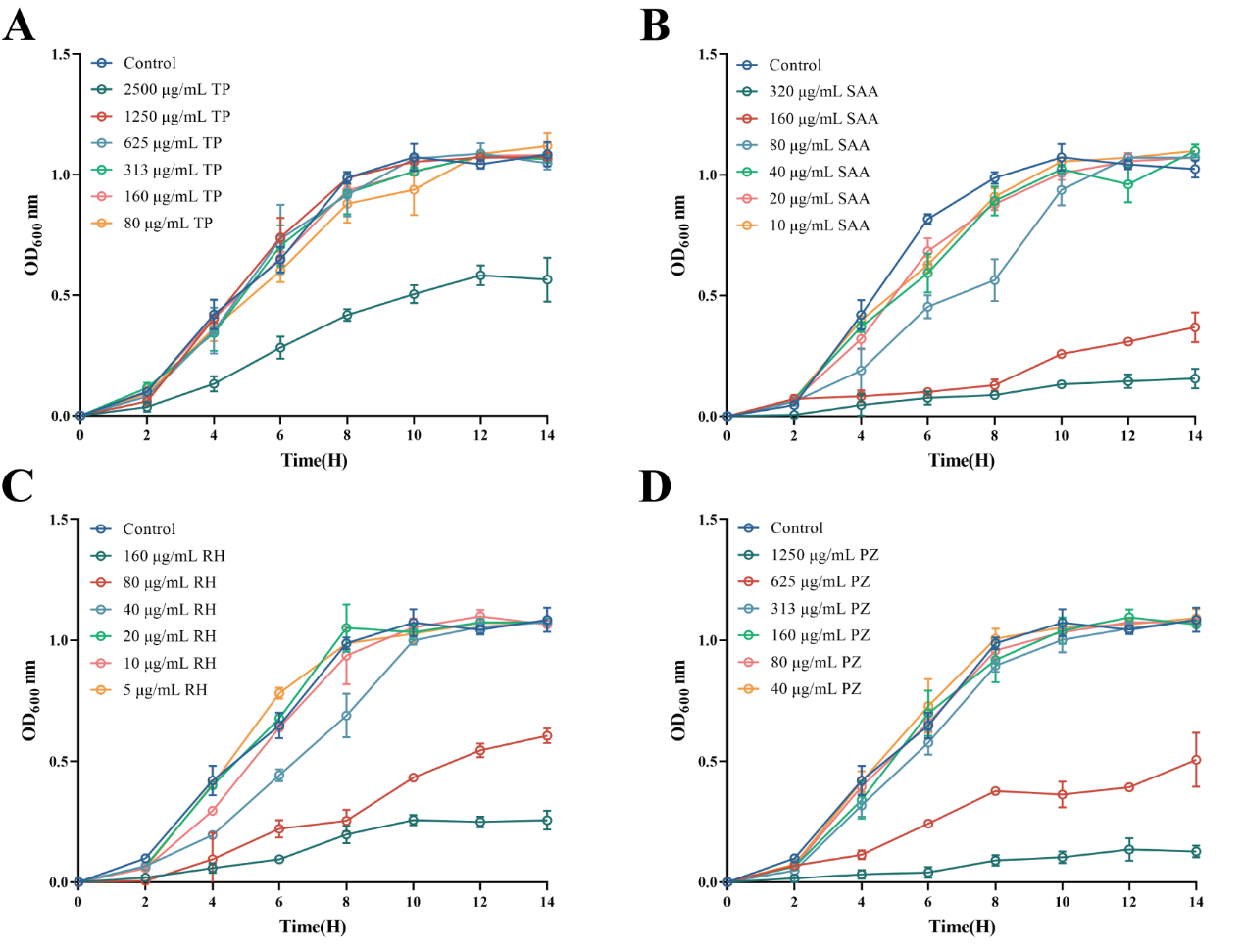


**Additional file 6. Growth curve of *S. suis* under the action of natural products.** (A) Growth of *S. suis* under the action of TP (A), SAA (B), RH (C) and PZ (D) respectively.
