# Supplementary material for: Targeting AI-2 quorum sensing: harnessing natural products against Streptococcus suis biofilm infection
Source: Vet Res. 2025 Feb 4;56:26. doi: 10.1186/s13567-025-01450-x (PMC11796197; doi:10.1186/s13567-025-01450-x)
Supplement: Supplementary file 7 — Additional file 7. Drug toxicity of natural products. [file 13567_2025_1450_MOESM7_ESM.docx]

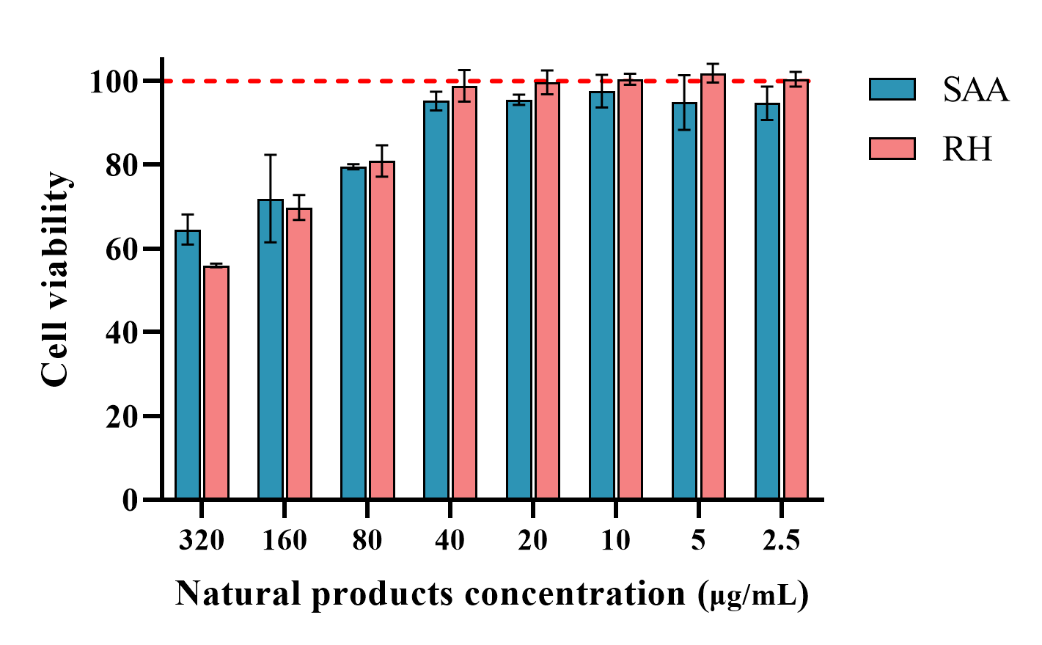


**Additional file 7 Drug toxicity of natural products.** Cell viability of HEp-2 under the action of SAA (A) and RH (B) respectively.
